# One-Pot Preparation of Cetylpyridinium Chloride-Containing Nanoparticles for Biofilm Eradication

**DOI:** 10.1021/acsabm.2c01080

**Published:** 2023-03-02

**Authors:** Alexander Brezhnev, Fung-Kit Tang, Chak-Shing Kwan, Mohammed S. Basabrain, James Kit Hon Tsoi, Jukka P. Matinlinna, Prasanna Neelakantan, Ken Cham-Fai Leung

**Affiliations:** † Restorative Dental Sciences, Discipline of Endodontology, Faculty of Dentistry, The University of Hong Kong, Hong Kong Island, Hong Kong SAR, P. R. China; ‡ Department of Chemistry, State Key Laboratory of Environmental and Biological Analysis, Hong Kong Baptist University, Kowloon Tong, Kowloon, Hong Kong SAR, P. R. China; § Applied Oral Sciences and Community Dental Care, Dental Materials Science, Faculty of Dentistry, The University of Hong Kong, Hong Kong Island, Hong Kong SAR, P. R. China; ∥ Division of Dentistry, The University of Manchester, Manchester M13 9PL, U.K.

**Keywords:** cetylpyridinium chloride, mesoporous silica nanoparticles, drug delivery, antimicrobial, biofilm eradication

## Abstract

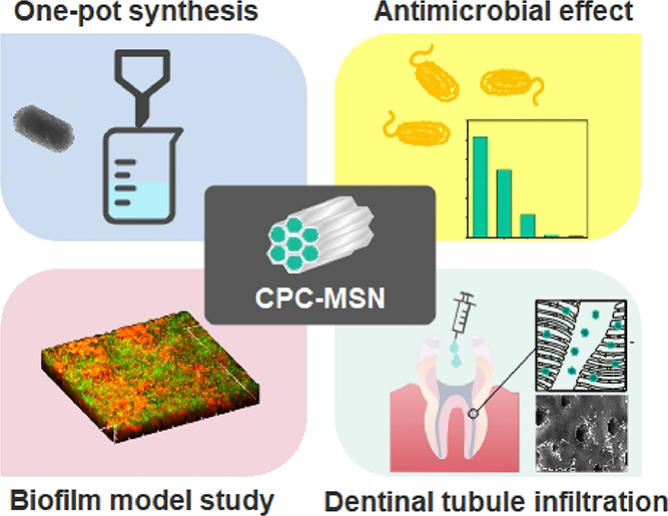

Quaternary ammonium compounds (QACs) have been widely used due to their excellent antimicrobial activity. However, using the technology where nanomaterials are employed as drug carriers to deliver QAC drugs has not been fully explored. In this study, mesoporous silica nanoparticles (MSNs) with short rod morphology were synthesized in a one-pot reaction using an antiseptic drug cetylpyridinium chloride (CPC). CPC-MSN were characterized via various methods and tested against three bacterial species (*Streptococcus mutans*, *Actinomyces naeslundii*, and *Enterococcus faecalis*), which are associated with oral infections, caries, and endodontic pathology. The nanoparticle delivery system used in this study prolonged the release of CPC. The manufactured CPC-MSN effectively killed the tested bacteria within the biofilm, and their size allowed them to penetrate into dentinal tubules. This CPC-MSN nanoparticle delivery system demonstrates potential for applications in dental materials.

## 1 Introduction

Recently, nanomaterials have been widely researched for the possibility of their usage for drug delivery, catalysis, and energy sources.^1,2^ A series of antibacterial drug-encapsulated nanomaterials have been investigated and proven to be successful drug delivery systems.^3−6^ In particular, hollow materials like mesoporous silica nanoparticles (MSNs) have attracted significant attention for their potential to carry an antimicrobial drug.^7−11^ Furthermore, they provided a high drug-loading capacity when used to carry benzalkonium chloride (BAC) against both *Staphylococcus aureus* and *Salmonella enterica*.^11^ BAC-containing MSNs exhibited a unique quality of pH-dependent release. Antimicrobial-loaded MSNs were incorporated into multiple dental materials, for example, a glass ionomer cement^12^ and dental adhesives,^13,14^ and tested against oral bacterial biofilms.^5,6^

Cetylpyridinium chloride (CPC) belongs to a quaternary ammonium compound (QAC) group of chemicals. It is a cationic surfactant with a broad-spectrum antimicrobial activity against bacteria and fungi.^15^ CPC’s antimicrobial ability is attributed to its positive charge (pyridinium cation), which allows it to adhere to microbial membranes, leading to disorganization and cell lysis.^15,16^ CPC is commonly used in different fields for its antimicrobial, antiviral, and antiplaque properties. Dentistry is not an exception since CPC is a component of multiple dental products such as mouth rinses, lozenges, dentifrices, and toothpastes.^17,18^ CPC is generally considered safe under certain conditions by the FDA^8,19,20^ and used for treating oral diseases and conditions such as gingivitis,^21^ dental plaque,^22^ and periodontitis.^23^ Likewise, incorporating CPC into dental restorative materials and root canal sealers can improve their antibacterial properties.^24,25^ The sustained release over a long period can enhance CPC’s antimicrobial potential. Recently, a hybrid CPC–montmorillonite composite material was successfully tested to prevent dental caries through its gradual release and rechargeability.^26^ CPC-containing sustained-release filler enables an effective release of the drug that can be used in root canal treatment.^27,28^

**Scheme 1:**
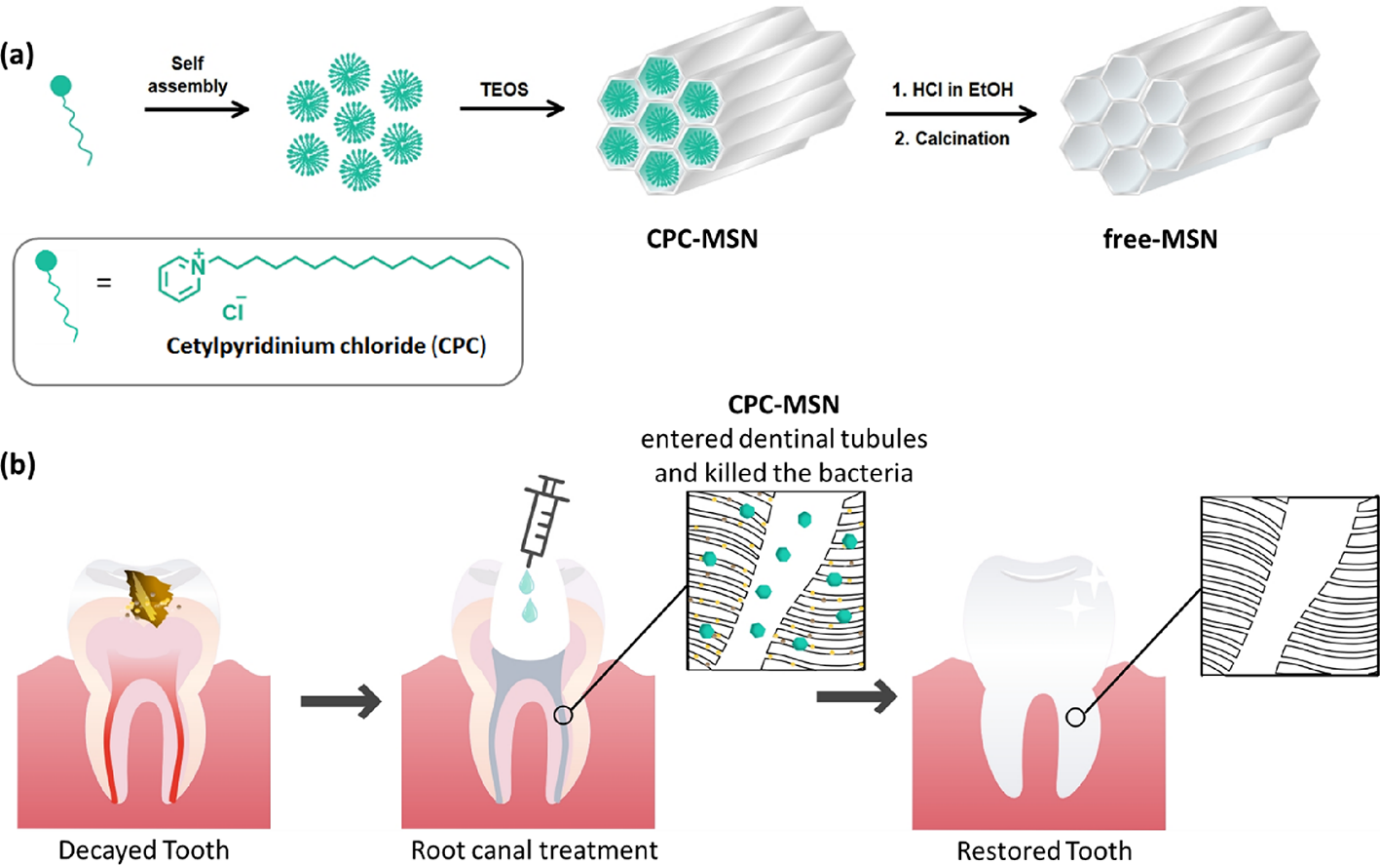
(a) Scheme of the Synthesis of CPC-MSN and Free-MSN. (b) Illustration of the Treatment of Biofilm Bacteria using CPC-MSN to Release the Drug

Previous studies attempted several approaches to employ CPC as a template to synthesize MSNs under various conditions.^11,29−31^ Yet, the requirements may involve other organic solvents and mixed surfactants, resulting in relatively lengthy synthetic procedures limiting the further biological applications. Therefore, these research works have drawn our attention to develop a better CPC drug delivery system. A typical synthesis of drug-encapsulated MSNs involves at least two to three steps. First, MSNs are produced through the template synthesis and undergo a series of washing steps. Second, drug loading into the pores of the MSNs is carried out using a highly concentrated drug solution. The loading process is driven by simple diffusion and physical adsorption on silica surfaces. Hence, the loading efficiency of a drug may be variable, and there is a high chance of trapping some organic solvents used in the drug loading process. To address this, a one-pot synthesis of MSNs using antibacterial drugs is cost-effective and enables a high-loading content without time-consuming preparation steps. In this study, CPC was directly employed as the drug template for the formation of the nanomaterial CPC-MSN, which was tested for its antimicrobial effect against biofilms and on the ability of this material to penetrate inside dentinal tubules (Scheme 1).

## 2 Materials and Methods

### 2.1 Materials

All chemicals and reagents, with the exception of the ones that were specified in the text, were purchased from Sigma Aldrich and used as received. The QAC used in the present study is cetylpyridinium chloride (CPC; 1-hexadecylpyridinium chloride). CPC was purchased from Sigma Aldrich (St. Louis, MO, USA). All solvents were used directly without further treatment or distillation.

### 2.2 Synthetic Procedures of CPC-MSN and Free-MSN

In general, CPC (0.50 g) was dissolved in deionized water (180 mL) at room temperature, and 25% ammonia water (8 mL) was slowly added as a base catalyst. The solution was mechanically stirred at 250 rpm, and TEOS solution (1.5 mL in 10 mL of EtOH) was then added dropwise from an additional funnel over 10 min. After reacting overnight, the solid suspension was transferred to the centrifuge tubes and the MSNs were isolated by centrifugation at 4000 rpm for 10 min. The particles were further washed with deionized water three times and EtOH three times with centrifugation. The resultant MSNs were dried in vacuum to obtain the product CPC-MSN as white powder. For comparison with the drug-free version, CPC-MSN was suspended in 10% HCl in EtOH and the mixture was refluxed for 24 h. The particles were collected by centrifugation and washed with deionized water three times with centrifugation. The particles were dried in vacuum and then calcinated at 500 °C for 4 h using a Thermolyne benchtop muffle furnace to obtain the free-MSN product as white powder. The products were characterized using microscopy techniques.

### 2.3 Characterization Methods

Infrared spectroscopy: IR spectra were recorded using a Perkin Elmer FT-IR Spectrum Two with an attenuated total reflection (ATR) detector. Powder X-ray diffraction measurement: PXRD analysis was performed using a Bruker D8 Advance X-ray diffractometer with a Cu Kα source. Thermogravimetric analysis: The thermal analysis of mass was performed using a Thermogravimetric Analyzer TGA-6 (PerkinElmer, USA). The heating rate was set to be 10 °C min^–1^ in the range from 100 to 800 °C. Dynamic light scattering: DLS of the sample was recorded with a DelsaMax CORE Light Scattering Analyzer (Beckman Coulter, USA) for the analysis of the particle size in solution medium. NMR spectroscopy: NMR spectra were recorded from a Bruker Advance–III 400 NMR spectrometer operating at 400 MHz for ^1^H and 101 MHz for ^13^C{^1^H}. Chemical shifts are reported in ppm. ^1^H and ^13^C chemical shifts were referenced internally with solvent residue chemical shift values (CDCl_3_: ^1^H, 7.26 ppm; ^13^C, 77.16 ppm). NMR data were processed using MestReNova Software (Mestrelab). UV–Vis absorption spectroscopy and release profile: UV–Vis absorption spectra were recorded using an Agilent Cary 8454 UV–Vis Spectrometer. Solution samples were contained in quartz cuvettes with a volume of 1.5 mL, 1 cm path length, and 0.4 cm slit length. All aqueous solutions were prepared with Milli-Q water (18.2 MΩ cm^–1^). CPC-MSN (100 mg) were dissolved in deionized water (50 mL) in a screwed flask. The mixture was stirred continuously, and 1 mL of solution was sampled and filtered at different time points: 1 min, 2 min, 5 min, 10 min, 20 min, 30 min, 40 min, 50 min, 60 min, 70 min, 80 min, 90 min, 100 min, 110 min, 120 min, 130 min, 140 min, 150 min, 160 min, 170 min, 180 min, 240 min, 24 h, and 48 h. The drug release content of the solution was then recorded in terms of absorbance. Electronic microscopy: TEM images were collected using a FEI Tecnai G2 20 S-TWIN Transmission Electron Microscope, and the SEM images were collected using a LEO 1530 Field Emission Scanning Electron Microscope, Hitachi S-4800 FEG Scanning Electron Microscope, and Hitachi SU1510 Microscope.

### 2.4 Bacterial Strains and Culture Conditions

*Streptococcus mutans* ATCC 700610, *Actinomyces naeslundii* ATCC 12104, and *Enterococcus faecalis* ATCC 29212 (American Type Culture Collection, Manassas, VA, USA) were acquired from the Department of Oral Biosciences, Faculty of Dentistry, The University of Hong Kong. Bacteria were taken from a freezer at −70 °C. The cells were spread with a sterile inoculating loop over the horse blood agar plates using a streak method. All the agar plates were placed in incubators for 48 h (anaerobic incubator for *S. mutans* and *A. naeslundii* and aerobic incubator for *E. faecalis*). Overnight cultures were prepared in Brain Heart Infusion (BHI) broth by inoculating colonies collected from horse blood (Hemostat Laboratories, Dixon, CA, USA) agar plates (Oxoid, Thermo Fisher Scientific). Incubation was performed in an anaerobic chamber with 5% CO_2_, 10% H_2_, and 85% N_2_ for *S. mutans* and *A. naeslundii* and aerobic chamber for *E. faecalis* at 37 °C. The cells were centrifuged at 5000 rpm for 10 min, washed twice in sterile phosphate-buffered saline (PBS, pH 7.4), and resuspended in fresh sterile BHI broth.

### 2.5 Assessment of Antimicrobial Activity: Determination of Minimum Inhibitory Concentration (MIC)

The antimicrobial susceptibility of bacteria to CPC-MSN was tested by determining the minimum inhibitory concentration by a broth microdilution assay following the guidelines of the Clinical and Laboratory Standards Institute.^32^ Planktonic suspensions (10^6^ CFU/mL) were added to wells of 96-well flat-bottom polystyrene cell culture microplates containing two-fold serially diluted CPC-MSN (2 to 128 μg/mL) in BHI. After incubation for 24 h at 37 °C anaerobically for *S. mutans* and *A. naeslundii* and aerobically for *E. faecalis*, the MIC was determined as the lowest concentration of CPC-MSN where complete inhibition of visible growth was observed. The assay was conducted three times in triplicate.

### 2.6 Assessment of Antimicrobial Activity: Determination of Minimum Bactericidal Concentration (MBC)

The MBC was determined by transferring 10 μL on horse blood agar plates from wells with CPC-MSN concentrations equal to and two times and four times higher than the MIC (wells that showed a complete absence of growth). The plates were incubated at 37 °C for 48 h under anaerobic (*S. mutans*, *A. naeslundii*) or aerobic (*E. faecalis*) conditions. The MBC was defined as the lowest concentration where no visible bacterial colonies were observed.^33^ The assay was conducted three times in triplicate.

### 2.7 Assessment of Antibiofilm Activity by an XTT Assay: Eradication of Preformed Biofilms

Minimum biofilm eradication concentrations (MBECs) of CPC-MSN were determined by evaluating biofilm metabolic activity using an XTT assay in 96-well flat-bottom polystyrene cell culture plates. For biofilm growth, bacteria were used in a concentration of 10^7^ CFU/mL in BHI supplemented with 0.2% sucrose.^34^ A total of 200 μL of bacterial suspension was added into wells of a 96-well plate for mono-species biofilm growth (*S. mutans*, *A. naeslundii*, and *E. faecalis*). After 24 h, the formed biofilms were washed gently with PBS twice. CPC-MSN were dispersed in BHI + 0.2% sucrose media and ultrasonicated for 1 min to get good dispersion of the particles in media at 1024 μg/mL concentration. Different concentrations (from 16 to 256 μg/mL) were further prepared and added into the wells (200 μL/well). Wells without compounds served as a growth control, while culture medium was used as a sterility control. The plates were incubated for 24 h at 37 °C anaerobically for *S. mutans* and *A. naeslundii* and aerobically for *E. faecalis*. The supernatant was discarded, and wells were washed gently with PBS twice. For biofilm metabolic activity evaluation, a freshly prepared reaction solution of PBS, 1 mg/mL XTT (2,3-bis(2-methoxy-4-nitro-5-sulfo-phenyl)-2*H*-tetrazolium-5-carboxanilide), and menadione (70 μg/mL) was used at a ratio of 79:20:1. A total of 200 μL of this reaction solution was added to the wells and left in the dark for 3 h at 37 °C.^35^ Then, the plates were centrifuged at 3000 rpm for 5 min. The supernatant (100 μL) was transferred to a new 96-well microplate, and the optical density was read at 492 nm (Spectra Max M2, Molecular Devices LLC, San Jose, CA, USA). MBEC was defined as the lowest concentration to provide at least 90% reduction in metabolic activity compared to the untreated control. The tests were performed in three unrelated experiments in triplicate. MSNs without CPC (free-MSN) were also tested following the same protocol.

### 2.8 Confocal Laser Scanning Microscopy (CLSM) Imaging of Biofilms

Single-species biofilms of *S. mutans* and *E. faecalis* were developed on sterile hydroxyapatite (HA) discs (diameter = 5 mm, thickness = 2 mm) (Clarkson Chromatography Products, Williamsport, PA, USA) by placing each HA disc in a separate well of sterile 24-well polystyrene plates (Nunc Thermanox TM, Thermo Fisher Scientific, Waltham, MA, USA) with inoculation media, 2 mL of BHI supplemented with 0.2% sucrose, containing 10^7^ CFU/mL *S. mutans* or *E. faecalis*. The plates were left in the anaerobic chamber for *S. mutans* (85% N_2_, 10% H_2_, 5% CO_2_) or aerobic chamber for *E. faecalis* at 37 °C for 24 h. After that, CPC-MSNs were dispersed in BHI supplemented with 0.2% sucrose to achieve a concentration of 1024 μg/mL and ultrasonicated for 1 min to get a good suspension of the particles, and further dilutions were prepared. The HA discs with biofilms were dip-washed twice in sterile PBS to remove the non-adherent cells and transferred to the following 2 mL treatment solutions in 24-well plates: Group 1: 128 μg/mL CPC-MSN, Group 2: 256 μg/mL CPC-MSN, Group 3: 512 μg/mL CPC-MSN, Group 4: control without treatment. After 24 h treatment, the HA discs were dip-washed twice in sterile PBS and transferred to 400 μL/well (in a 48-well plate) stain of a LIVE/DEAD BacLight Bacterial Viability Kit (SYTO 9/propidium iodide, 1:1 solution in DMSO) L7012 (Invitrogen Detection Technologies, USA) for 30 min and left at room temperature in the dark. Then, the HA discs were transferred to coverslips and examined at five random points by the oil-immersion objective lens (×60) of a confocal laser scanning microscope (CLSM) (Fluoview FV 1000, Olympus, Tokyo, Japan), a two-photon laser scanning microscope. 3D images of the biofilms were reconstructed from Z-stacks with Olympus Fluoview software (FV10-ASW 4.2 Viewer), and architecture of biofilms was observed. Calculation of the percentage ratio of live/dead cells was performed by Bioimage_L v.3.0 software.^36^

### 2.9 CCK-8 Cytotoxicity Assay

NIH/3T3 mouse fibroblasts^37^ (from the stock in the tissue culture lab, Faculty of Dentistry, The University of Hong Kong) were cultured in Dulbecco’s Modified Eagle Medium (DMEM) (Thermo Fisher Scientific, Waltham, MA) with high glucose supplemented with 10% fetal bovine serum (Thermo Fisher Scientific, Waltham, MA), 1% of 100 U/mL penicillin, and 100 μg/mL streptomycin (Thermo Fisher Scientific, Waltham, MA) under standard cell culture conditions (37 °C, 100% humidity, 95% air, and 5% CO_2_). 100 μL per well of 96-well plates, NIH/3T3 cells were seeded at a density of 2 × 10^4^ cells per well (2 × 10^5^ cells/mL). The plates were incubated for 24 h to provide cell attachment. CPC-MSN and free-MSN were dispersed in DMEM to prepare 1024 μg/mL concentration and ultrasonicated for 1 min, and further two-fold serial dilutions were prepared. After incubation and removal of media, 100 μL of each corresponding concentration was added to the wells with cells and incubated for 24 h. Cell viability was determined by a Cell Counting Kit-8 assay (CCK-8) (Dojindo Laboratories, Kumamoto, Japan). After treatment, the supernatant was aspirated gently from the wells, and 100 μL of DMEM with 10 μL of CCK-8 was added into each well. After incubation for 2 h, the plates were centrifuged at 3000 rpm for 5 min, and 85 μL of the supernatant from each well was transferred to a new 96-well plate. The optical density was read at 450 nm wavelength with a spectrophotometer (SpectraMax M2, Molecular Devices, LLC, San Jose, CA, USA). Cells in DMEM (without treatment) served as the control group. As a blank control, wells with DMEM media without cells or MSNs were used. The average of this absorbance value was subtracted from the absorbance value of each well. The data was normalized to the control, and the percentage of viable cells was calculated. The tests were performed on three different occasions in triplicate.

### 2.10 Preparation of Teeth Samples and Infiltration of CPC-MSN into Dentinal Tubules

Two extracted permanent premolars were cleaned by removing the soft tissues. The teeth were further stored in 1:1 solution of 0.9% saline and 0.5% thymol at 4 °C.^38^ A slow-speed diamond impregnated saw (Isomet 5000, Buehler, Lake Bluff, IL) was used to remove the crowns of the teeth with water cooling to obtain the roots.^38^ Teeth were cut into two longitudinal halves through the root canals. To remove organic remnants such as bacteria and pulp tissue, the samples were immersed in 5.25% NaOCl for 5 min and washed with deionized water. After that, the samples were immersed in 17% EDTA for 5 min and washed with deionized water again. CPC-MSN were dispersed in distilled water and ultrasonicated for 1 min to prepare a 256 μg/mL suspension. The samples were immersed into the suspensions of CPC-MSN and ultrasonicated for 5 min. They were allowed to dry, mounted to a SEM stub, sputter-coated with palladium and platinum, and examined by SEM (SU1510, Hitachi, Tokyo, Japan).

### 2.11 Statistical Analysis

All experiments were performed at least in triplicate on three different occasions. Statistical analysis was performed using one-way ANOVA and two-way ANOVA followed by a Tukey’s HSD or SNK post-hoc test. The statistical significance was set at *p* < 0.05. Data were presented as mean ± standard deviation (SD). Statistical analysis was performed with SPSS software (version 26, Chicago, IL).

## 3 Results and Discussion

First, we screened several concentrations for template synthesis of the MSNs using a sol–gel method. In brief, CPC self-assembles to form uniform micelles under ultrasonication in a water–alcohol medium. Organosilicon reagent tetraethyl orthosilicate (TEOS) was added dropwise to promote hydrolysis and polycondensation to form the mesoporous silica structure eventually. The obtained solid was washed and dried to collect the CPC-MSN product. As a control for comparison, the surfactant from CPC-MSN was washed with a hot acidic alcoholic solution and then water and treated at a high temperature (calcination) to entirely remove the organic matter to get the drug-free version, free-MSN (Scheme 1a).

**Figure 1:**
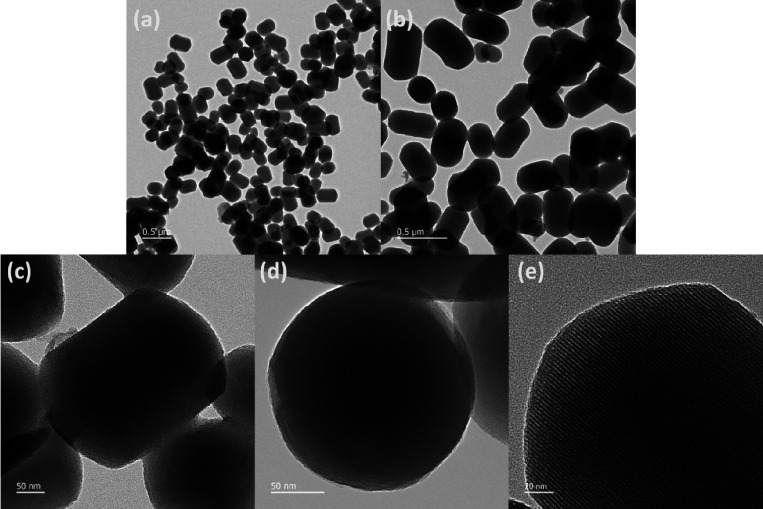
Transmission electron microscopy images (TEM) of CPC-MSN. Scale bar: (a, b) 500 nm, (c, d) 50 nm, and (e) 20 nm.

**Figure 2:**
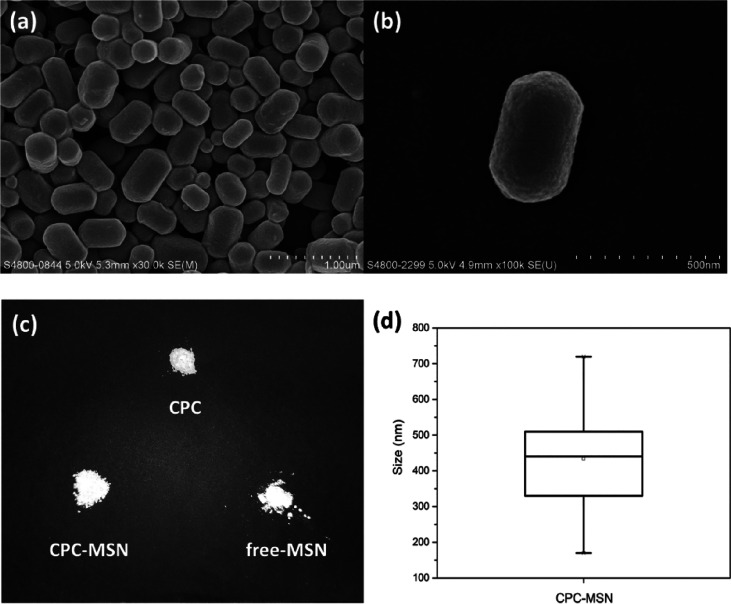
(a, b) Scanning electron microscopy (SEM) images of CPC-MSN. (c) Appearance of CPC, CPC-MSN, and free-MSN. (d) Box-plot showing the size distribution of CPC-MSN.

The synthesized CPC-MSN were characterized by both transmission electron microscopy (TEM) and scanning electron microscopy (SEM), which showed regular-sized mesoporous nanoparticles featuring short rod, cylindrical morphology with hemispherical ends (Figure 1a–d and Figure 2a,b). The estimated mean size was approximately 426 nm in length and 261 nm in width (Figure 2d and Figure S3), and thereby the average aspect ratio was determined as 1.63. The pore diameter was estimated from the *d*-spacing values using the fast Fourier transform (FFT) of the HR-TEM, and it suggested that the pore size of CPC-MSN is 3.06 nm ± 0.09 nm (Figure 1e and Figure S4). In small-angle X-ray powder diffraction (SAXRD) analysis, the peaks at 2θ = 2.41, 4.14, 4.75, and 6.31 could be correlated to the (100), (110), (200), and (210) reflections, which suggested the hexagonal symmetry unit cell in CPC-MSN, and the results were similar to the reported literature (Figure S5).^29,39^ In wide-angle X-ray powder diffraction (WAXRD) analysis, the broad diffraction peak with a Bragg angle at 2θ = 22.4° suggested the amorphous silica nature of the synthesized CPC silica nanoparticles (Figure S6).^39^ The physical appearance of CPC-MSN, free-MSN, and pure CPC is white powder (Figure 2c) with only pristine CPC showing crystallinity, but they have different chemical composition in the following analysis. In addition, the white appearance fits the color scheme of filling materials, providing great potential for use in dental materials such as resin composites, adhesives, root canal materials, etc. in our future studies.

**Figure 3:**
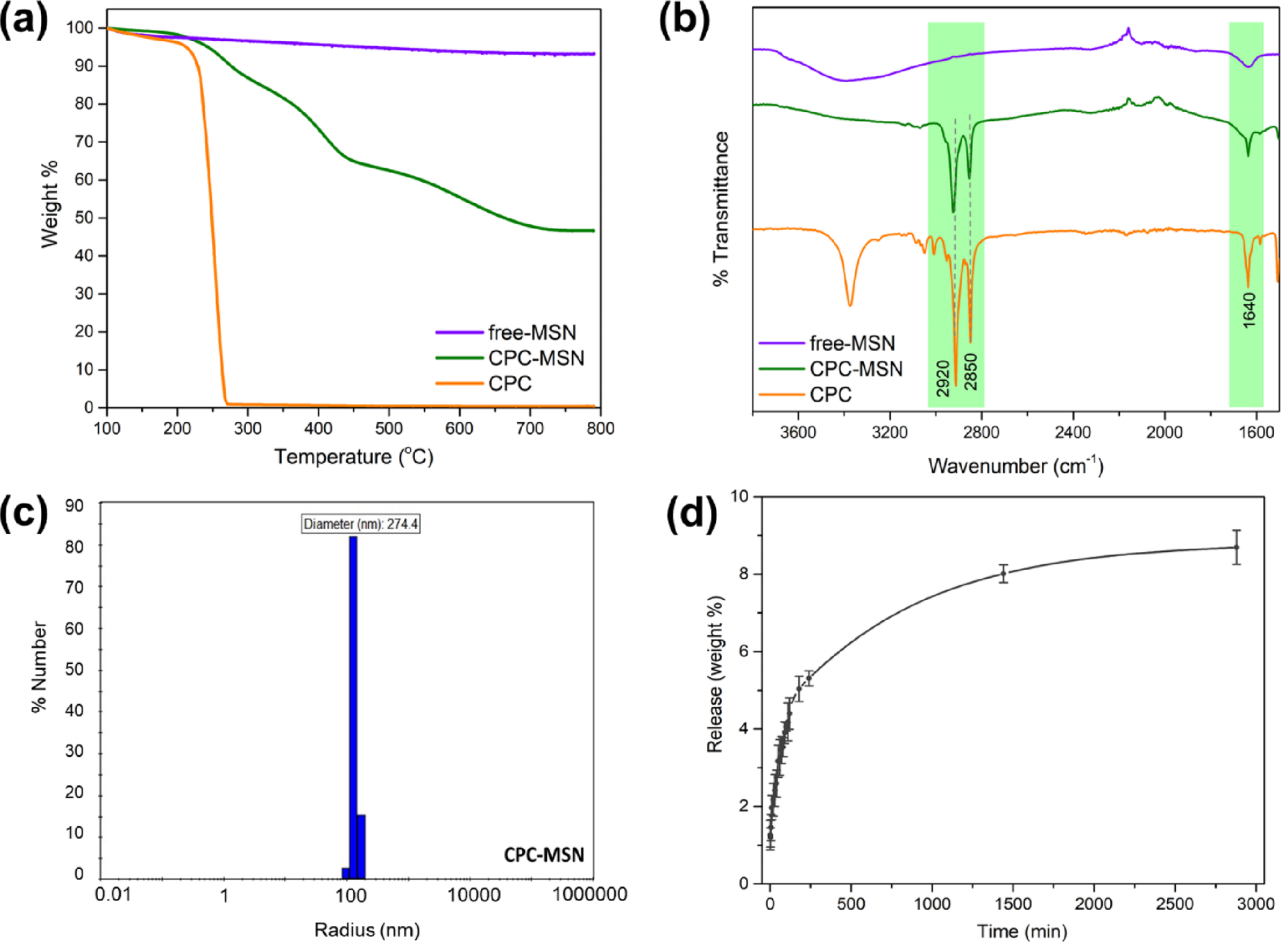
(a) Thermogravimetric analysis (TGA) curves of CPC-MSN, free-MSN, and pristine CPC. (b) Stacked infrared (IR) spectra of CPC-MSN, free-MSN, and pristine CPC. (c) Dynamic light scattering (DLS) measurement of CPC-MSN in aqueous medium. (d) Release profile of CPC from CPC-MSN in aqueous medium.

The loading efficiency of CPC-MSN was estimated by thermogravimetric analysis (TGA), where the weight loss above 230 °C was caused by the thermal degradation of CPC drug encapsulated in the nanoparticles (Figure 3a). The weight percentage loading of the drug is about 47% ± 3% (0.89 g per 1 g of SiO_2_), which is estimated from the weight loss of CPC-MSN ranging from 230 to 800 °C. The thermal decomposition of CPC-MSN was stepwise compared to a sharp decomposition of pristine CPC, and the difference could be attributed to the strong interaction between the charged head groups and the silica surfaces, the presence of other ions, and the embedded drugs during synthesis of silica structures.^7,29^ Overall, the loading of CPC in CPC-MSN is high due to the one-pot template synthesis, and these drug nanocarriers may be beneficial for dental applications in the long term. Infrared spectroscopy was also carried out to identify the functional groups of CPC in CPC-MSN. As shown in Figure 3b, the peaks at 2920 and 2820 cm^–1^ were observed corresponding to the C–H stretching of the alkyl chains of CPC, and the peaks at 1640 cm^–1^ were observed corresponding to the aromatic C=C stretching of the pyridinium ring. Both ν_(C–H)_ and ν_(C=C)_ frequencies were also present in CPC-MSN with high intensities but not in free-MSN, which suggests a good encapsulation of the drug content in the CPC-MSN nanocarriers. In addition, the hydrodynamic size of CPC-MSN in solution was measured to be around 274 nm in diameter by dynamic light scattering (DLS) (Figure 3c). Finally, the drug release of CPC-MSN was measured by UV–Vis absorption spectroscopy in water medium and is presented in weight % release (Figure 3d). There was a burst release in the first 3 h followed by a slow gradual release, reaching the plateau at around 8–9 wt % after 48 h. All the data suggests that CPC-MSN nanocarriers have a high percentage loading of CPC drug. It can potentially be used as a prolonged drug release system in dental applications to prevent biofilm formation and fight preformed biofilms.

The choice of bacteria in this study was performed by taking into consideration a potential use of CPC-MSN against caries, for example, in dental adhesives and filling materials, and in endodontics. In this study, we tested the effect of CPC-MSN against three strains of Gram-positive oral bacteria. *E. faecalis* is often tested as a biofilm model bacteria in endodontics, in particular in root canal disinfection, and it is the most frequently tested species due to its clinical significance.^40^*E. faecalis* is often found in failed root canals and is commonly isolated from persistent endodontic lesions.^41,42^ It also takes part in the pathogenesis of a number of serious conditions.^43^*S. mutans* is associated with dental caries development.^44−46^ Moreover, *S. mutans* and *A. naeslundii* are found in caries and endodontic lesions.^47−49^

According to Costerton *et al.*, a biofilm is an aggregation of sessile microbial cells that are adhered to colonized surfaces and enveloped in an exopolysaccharide protective matrix.^50^ Biofilms can be described as a complex microenvironment consisting of microbes and supporting polymeric matrix containing extracellular DNA, proteins, polysaccharides, etc.^17^ The embedded bacteria are shielded, and there are several mechanisms to induce the antibiotic resistance.^51^ Therefore, new strategies are needed to overcome the problem of antimicrobial resistance, including the possibilities of nanotechnology, MSNs in particular.^52^ In dentistry, biofilm formation can lead to the development of endodontic pathology, periodontal diseases, caries, and many other conditions.

In our study, we tested CPC-MSN against both planktonic forms and biofilms of these three pathogens. For planktonic cells, the MIC and MBC results are shown in Table 1. 16 μg/mL CPC-MSN was required to inhibit the growth of planktonic bacteria. The minimum bactericidal concentration for all three planktonic bacteria was 32 μg/mL. Free-MSN (without CPC drug) even at a high concentration of 1024 μg/mL did not inhibit the growth of planktonic microorganisms, proving that the antibacterial effect is attributed to the released CPC drug.

**Table 1:** Minimum Inhibitory Concentration (MIC) and Minimum Bactericidal Concentration (MBC) of CPC-MSN (in μg/mL)

strains	MIC (μg/mL)	MBC (μg/mL)
*E. faecalis*	16	32
*S. mutans*	16	32
*A. naeslundii*	16	32

**Figure 4:**
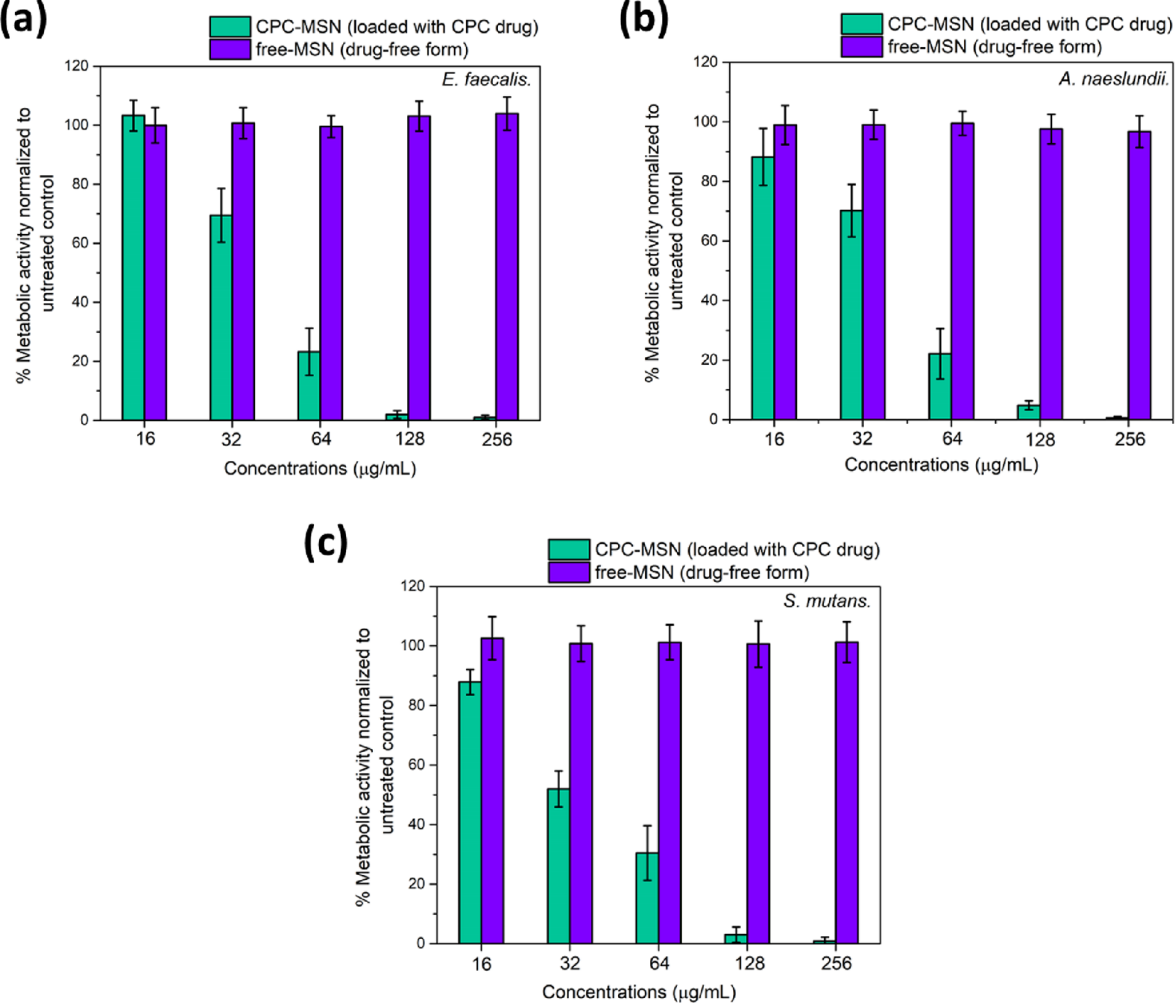
Metabolic activity of 24 h-old biofilms after treatment with CPC-MSN for 24 h. (a) *E. faecalis*, (b) *A. naeslundii*, and (c) *S. mutans.* The metabolic activity (expressed as percentage relative to the untreated control) was established using the XTT assay. OD readings were performed at 492 nm.

An XTT assay was conducted to test the bacterial viability by determining the biofilm metabolic activity of three single-species biofilms using increasing concentrations of CPC-MSN. The minimum concentrations to provide more than 90% reduction in biofilm metabolic activity in all three biofilms (MBECs) were established to be 128 μg/mL, and the values of bacterial viability among different concentrations ranging from 16 to 128 μg/mL were significantly different from each other (*p* < 0.05). It can be noted that drug-free MSNs (free-MSN) did not affect the metabolic activity, meaning that they were inactive toward biofilms in all concentrations tested with no significant difference among the groups (*p* > 0.05) (Figure 4a–c). It proves that the encapsulated CPC is crucial to the antibacterial properties of CPC-MSN.

**Figure 5:**
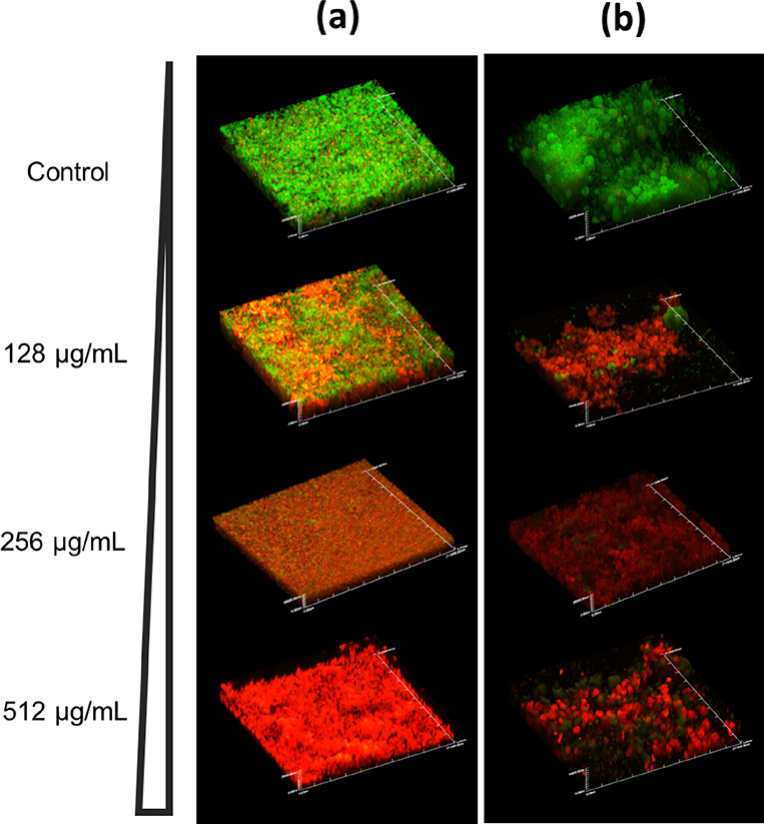
(a) Confocal laser scanning microscopy images (CLSM) of 24 h-old *E. faecalis* biofilms on HA discs after treatment with increasing concentrations of CPC-MSN for 24 h. (b) Confocal microscopy images of 24 h-old *S. mutans* biofilms on HA discs after treatment with increasing concentrations of CPC-MSN for 24 h. Magnification: ×60, scale = 210 μm. The green fluorescent dye (SYTO-9) labeled cells with intact membranes, while the red fluorescent dye (PI) labeled cells with damaged membranes.

In CLSM experiments, *E. faecalis* and *S. mutans* bacteria were grown as mono-species biofilms on hydroxyapatite (HA) discs and treated with CPC-MSN with the following concentrations: 128, 256, and 512 μg/mL. Our results showed that untreated control groups of both *E. faecalis* and *S. mutans* biofilms were mostly composed of live cells (labeled in green) with a few dead cells (labeled in red). In *E. faecalis* groups (Figure 5a), biofilms treated with 128 μg/mL consist of partially damaged (yellowish green), dead (red), and live (green) cells. Live/dead analysis of the images established that 128 μg/mL concentrations provided a mean ratio of 45.8% dead cells. When the concentration went up to 256 μg/mL, the amount of yellowish and red cells increased significantly and constituted the majority of the cells (mean ratio of 85%), while at 512 μg/mL, almost no live cells were observed (99.8% dead cells). There was some difference in the results of the XTT assay and CLSM for the treated *E. faecalis* biofilms, and this could be attributed to the different substrates for culturing of *E. faecalis*, such as HA discs for CLSM experiments. In *S. mutans* groups (Figure 5b), very few live cells were detected even at 128 μg/mL (89.9% dead cells), which is consistent with the XTT assay results. Concentrations of 256 and 512 μg/mL for *S. mutans* provided the mean ratio of dead cells of 99.8% in both groups. For both *E. faecalis* and *S. mutans*, all the treatment groups (drug concentrations) were statistically different from each other (with the exception of no difference between 256 and 512 μg/mL for *S. mutans*), and all treatment groups were different compared to untreated controls (*p* < 0.001). The concentration-dependent killing pattern is observed in both XTT and CLSM experiments. Our treatment exhibited antimicrobial effects and killed bacterial cells in biofilms.

Results from the CCK-8 assay demonstrated that the cytotoxicity of CPC-MSN was also due to the encapsulated CPC drug, while the drug-free version free-MSN showed no cytotoxicity up to 256 μg/mL with no adverse effect on the cell viability in all concentrations tested without difference among the groups (*p* > 0.05). In the drug-loaded groups, CPC-MSN at 16 μg/mL exhibited around 50% viability reduction compared to the untreated control, and at 32 μg/mL, all cells were not viable (Figure S8). The results were also supported by the observation of the cell shape, attachment, and appearance under the bright-field cell microscope, where live cells are observed as being of normal spindle-shaped morphology and attached to the wells, while dead cells are round-shaped and detached (Figure S9). Cytotoxicity results should be considered as a reference due to the fact that commonly used antiseptics (including chlorhexidine and CPC) possess a certain level of toxicity toward cells, although they are widely used in medical and dental fields. Moreover, Yang *et al.* reported on the possibility of the components of Dulbecco’s Modified Eagle Medium (DMEM) to erode the surface silica, which may lead to faster release of the loaded drugs, and results of the cytotoxicity may be overestimated.^53^

**Figure 6:**
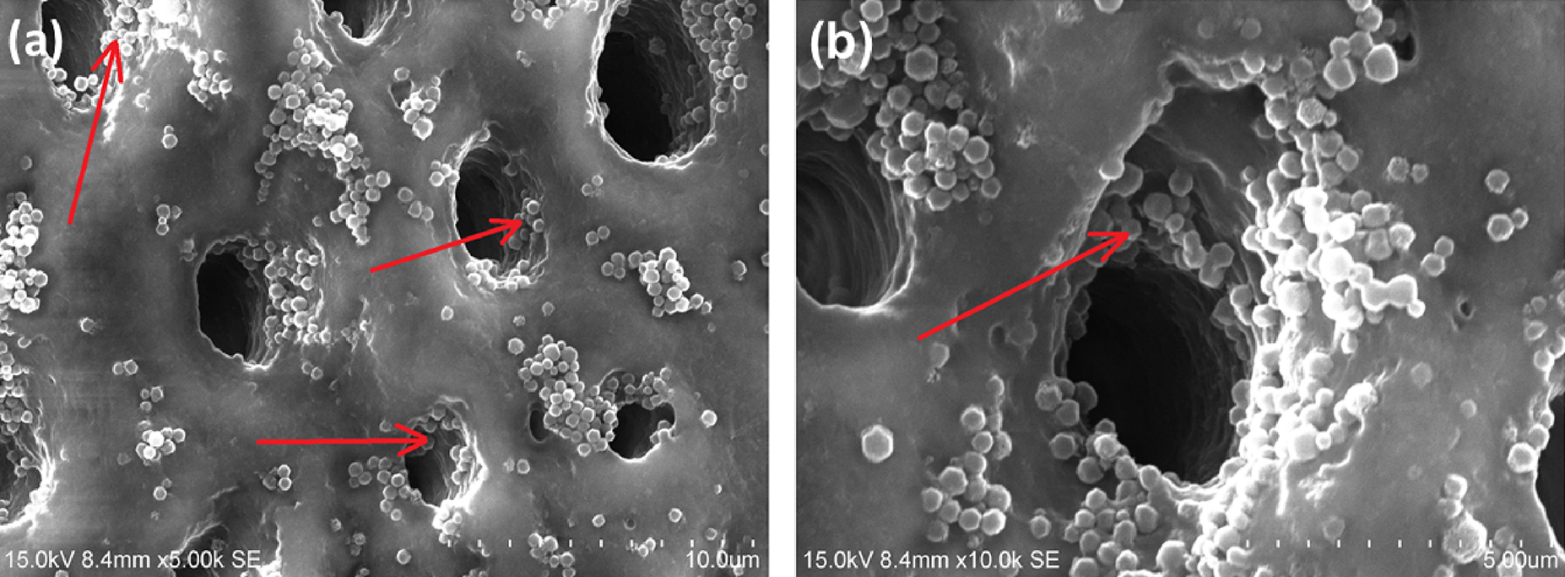
Scanning electron microscopy (SEM) images showing infiltration of CPC-MSN inside dentinal tubules. Arrows pointing to CPC-MSN inside dentinal tubules. Scale: (a) 10 μm and (b) 5 μm.

*E. faecalis* cells have the ability to invade dentinal tubules, attach to collagen, and stay in the form of biofilms in these root canal complexities.^54^ They may cause persistent infections. It leads to serious difficulties in solving such conditions. Therefore, for potential prevention and elimination of these bacteria, the size of our CPC-MSN nanocarriers has to be small enough to go inside these anatomical structures. Our preliminary results show that CPC-MSN can infiltrate and stay inside the dentinal tubules (Figure 6 and Figure S10), thereby achieving a continuous release of the encapsulated drug to eliminate the pathogens in the long term.

## 4 Conclusions

In conclusion, a mesoporous silica nanocarrier structure CPC-MSN was obtained through a one-pot reaction pathway with the antiseptic drug CPC, which is used to attain a high drug loading content for potential dental applications. CPC-MSN showed a remarkable antibacterial effect toward common dental pathogens, *S. mutans*, *A. naeslundii*, and *E. faecalis*, while free-MSN particles in tested concentrations were not active toward both bacterial and murine cells. The metabolic activity of these biofilms was affected by CPC-MSN in a dose-dependent manner. The mono-species biofilms of *E. faecalis* and *S. mutans* were formed on HA discs and treated with CPC-MSN, and the results confirmed its antibiofilm effect against the tested pathogens. All the results of our study demonstrated that CPC-loaded mesoporous silica nanocarriers have the advantage of high percentage loading, suggesting their potential application as drug delivery systems to combat biofilms and overcome numerous challenges such as delivery of drug molecules to intricate anatomical structures inside the tooth to provide the desired bactericidal properties. Studies on CPC-MSN for the prevention and treatment of caries and root canal infections may be beneficial. The particles may be incorporated in dental adhesives and filling materials and can be used for endodontic treatment. Research on multiple-species mature biofilm models with appropriate clinically relevant well-designed experiments will be carried out in the future.

## Supporting Information Available

TEM and SEM images of CPC-MSN; size distribution of CPC-MSN; X-ray diffraction spectra of CPC-MSN; UV–Vis spectrum of CPC in H_2_O; cell viability of NIH/3T3 mouse fibroblasts after treatment with CPC-MSN and free-MSN; SEM images for dentinal tubule penetration by CPC-MSN; bright-field cell images (NIH/3T3 cells) after treatment with CPC-MSN and free-MSN; ^1^H and ^13^C NMR spectra of pristine CPC used (PDF)
