# Supplementary material for: One-Pot Preparation of Cetylpyridinium Chloride-Containing Nanoparticles for Biofilm Eradication
Source: ACS Appl Bio Mater. 2023 Mar 2;6(3):1221–30. doi: 10.1021/acsabm.2c01080 (PMC10031565; doi:10.1021/acsabm.2c01080)
Supplement: Supplementary file 1 — mt2c01080_si_001.pdf [file mt2c01080_si_001.pdf]

Supporting Information

One-Pot Preparation of Cetylpyridinium Chloride-Containing Nanoparticles for Biofilm Eradication

Alexander Brezhnev,‡[a] Fung-Kit Tang,‡[b] Chak-Shing Kwan,[b] Mohammed S. Basabrain,[a] James Kit Hon Tsoi,[c] Jukka P. Matinlinna,[cd] Prasanna Neelakantan*[a] and Ken Cham-Fai Leung*[b]

[a] Restorative Dental Sciences, Discipline of Endodontology, Faculty of Dentistry, The University of Hong Kong, Hong Kong Island, Hong Kong SAR, P. R. China

[b] Department of Chemistry, State Key Laboratory of Environmental and Biological Analysis, Hong Kong Baptist University, Kowloon Tong, Kowloon, Hong Kong SAR, P. R. China

[c] Applied Oral Sciences and Community Dental Care, Dental Materials Science, Faculty of Dentistry, The University of Hong Kong, Hong Kong Island, Hong Kong SAR, P. R. China

[d] Division of Dentistry, The University of Manchester, Manchester, M13 9PL, United Kingdom

*E-mails: cfleung@hkbu.edu.hk (K.C.-F.L.); prasanna@hku.hk (P.N.)

‡ These authors contributed equally to this work.

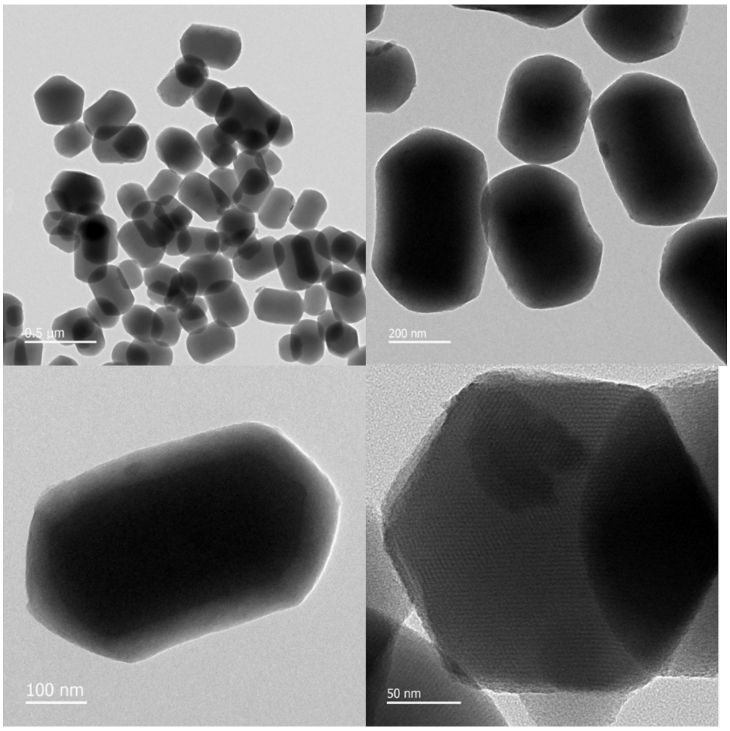

Figure S1. Additional transmission electron microscopic (TEM) images of **CPC-MSN** at different magnifications.

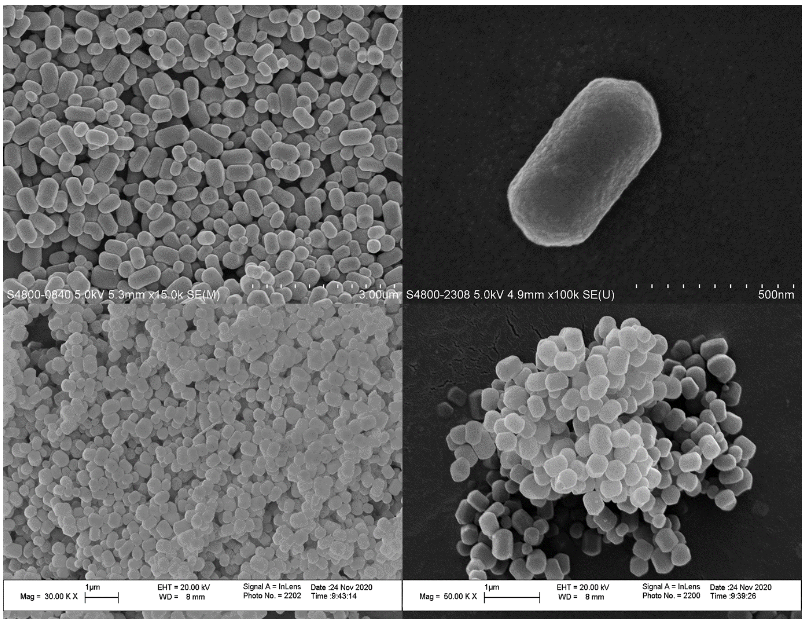

Figure S2. Additional scanning electron microscopic (SEM) images of **CPC-MSN** at different magnifications.

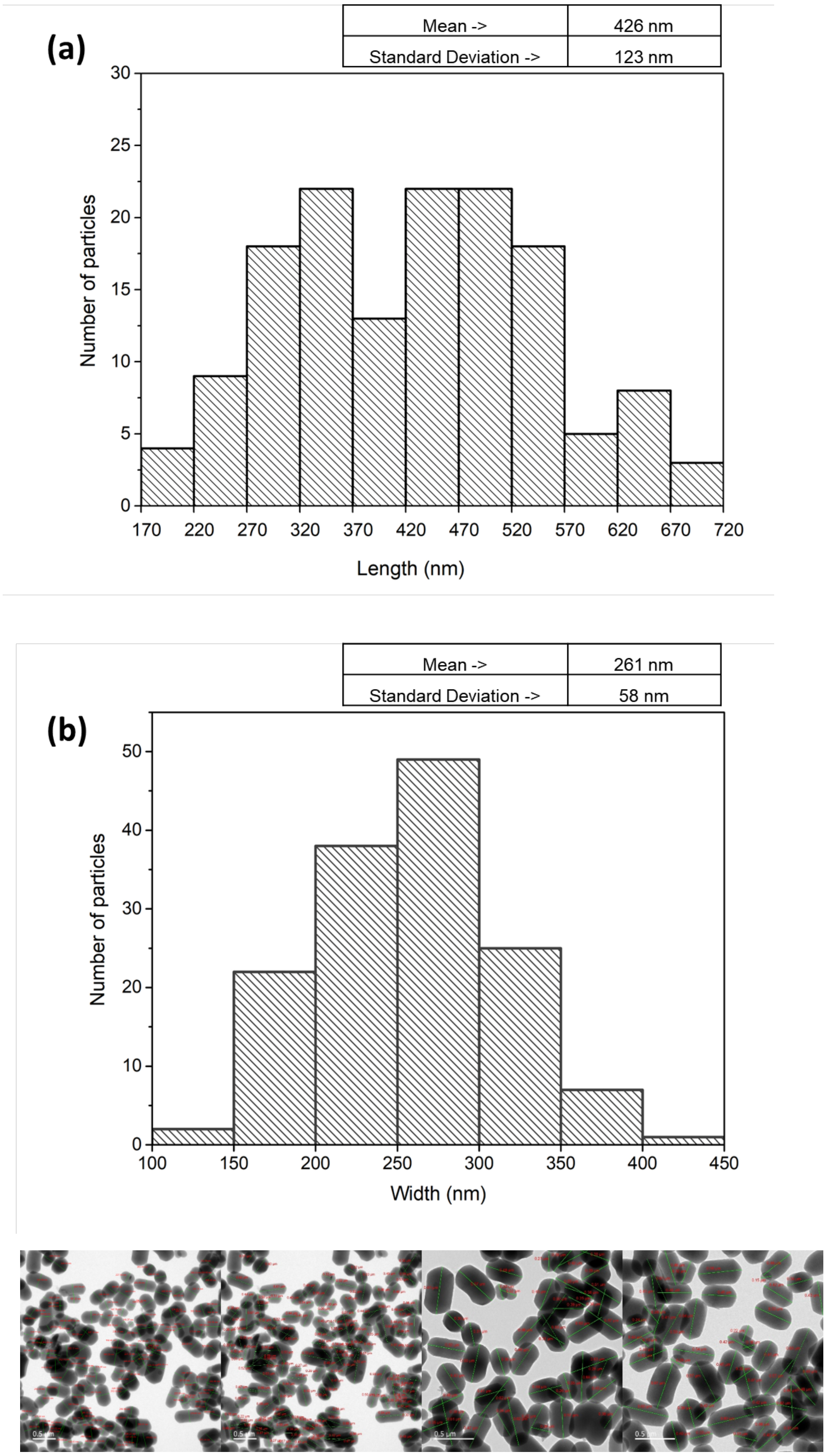

Figure S3. Size distribution of **CPC-MSN** (estimated from TEM images). (a) Length; (b) Width. Number of particles measured = 145.

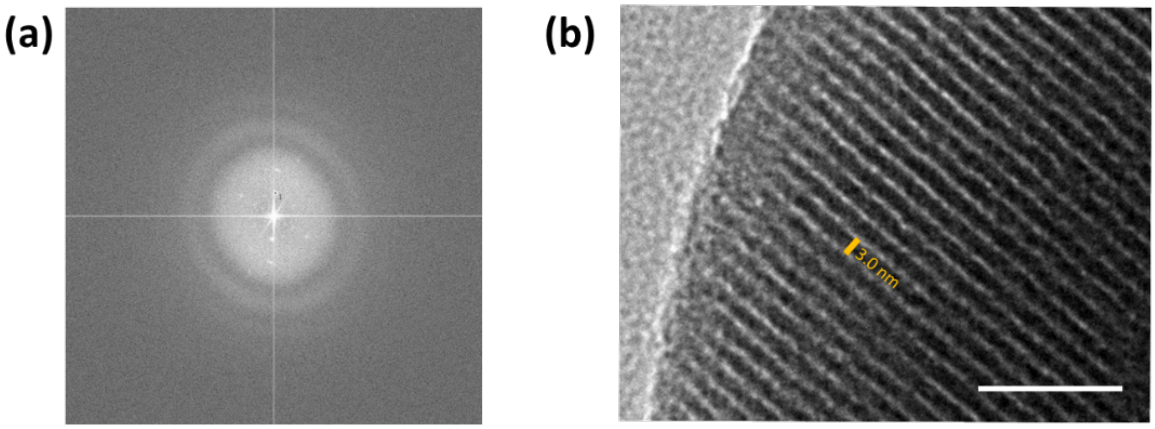

Figure S4. (a) Diffractogram of **CPC-MSN** through Fast Fourier Transform (FFT) analysis. (b) Enlarged TEM image showing the channels. Scale bar = 20 nm.

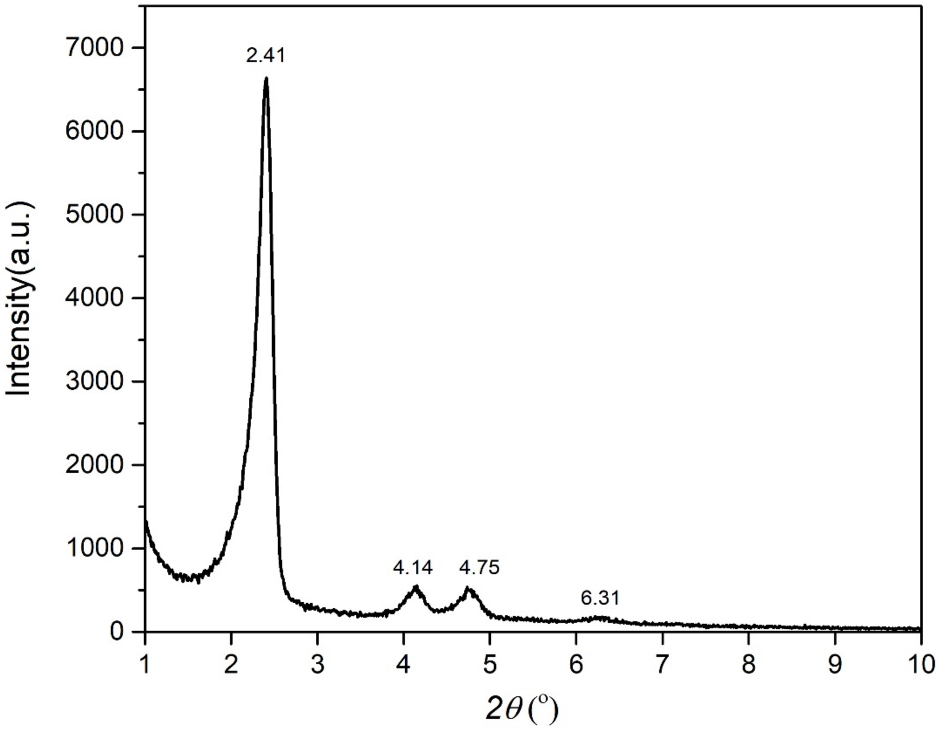

Figure S5. Small-angle X-ray powder diffraction (SAXRD) pattern of **CPC-MSN**.

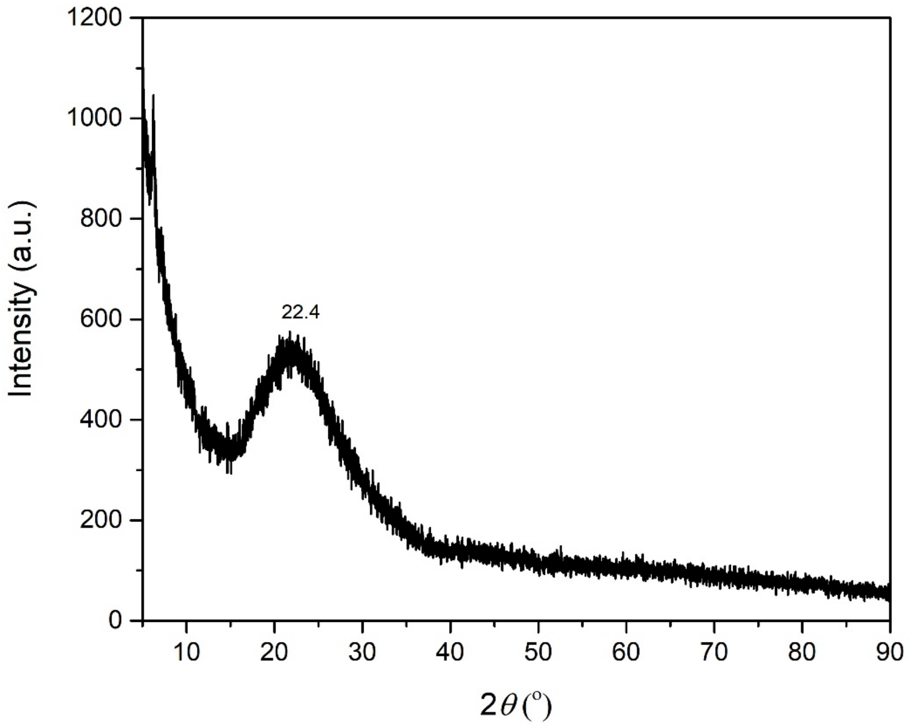

Figure S6. Wide-angle X-ray powder diffraction (WAXRD) pattern of **CPC-MSN**.

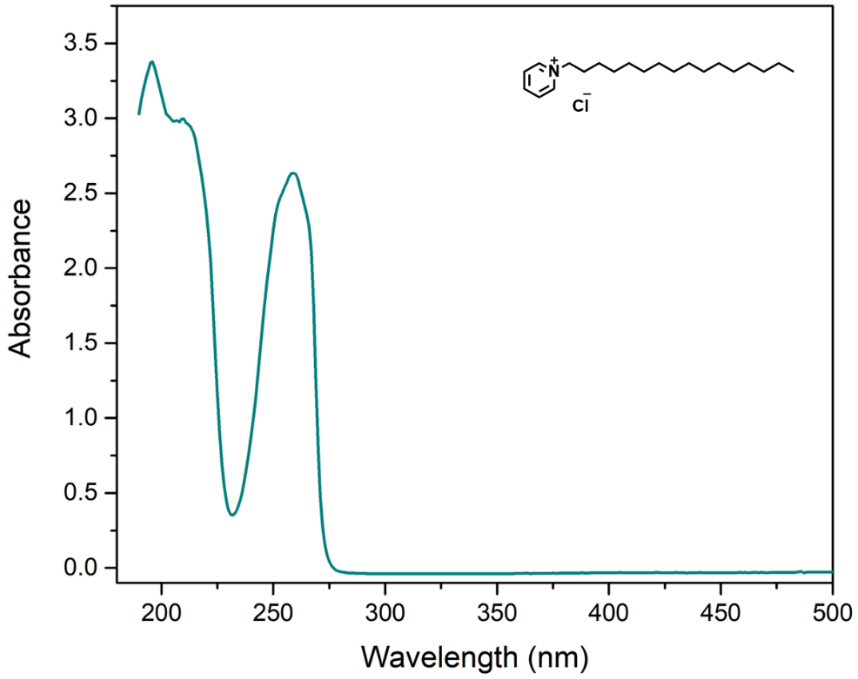

Figure S7. UV-Vis spectrum of 1 mM cetylpyridinium chloride (CPC) in H_2O. Molar extinction coefficient was estimated as 6573 M^{-1} cm^{-1} at 260 nm.

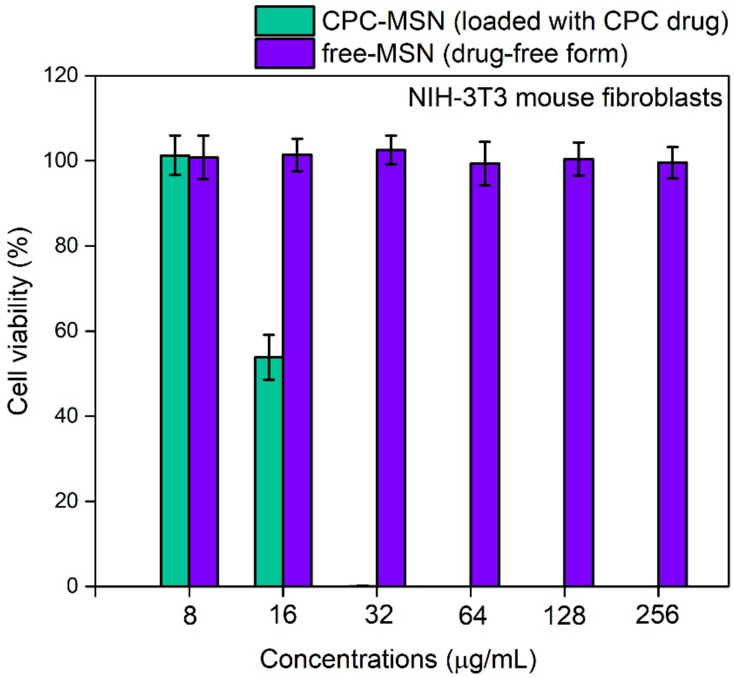

Figure S8. The cell viability of NIH/3T3 mouse fibroblasts after 24 h treatment with **CPC-MSN** (loaded with CPC drug) and **free-MSN** (drug-free form) was assessed using CCK-8 by measuring the absorbance of the supernatant at 450 nm wavelength. Three independent experiments in triplicate were performed.

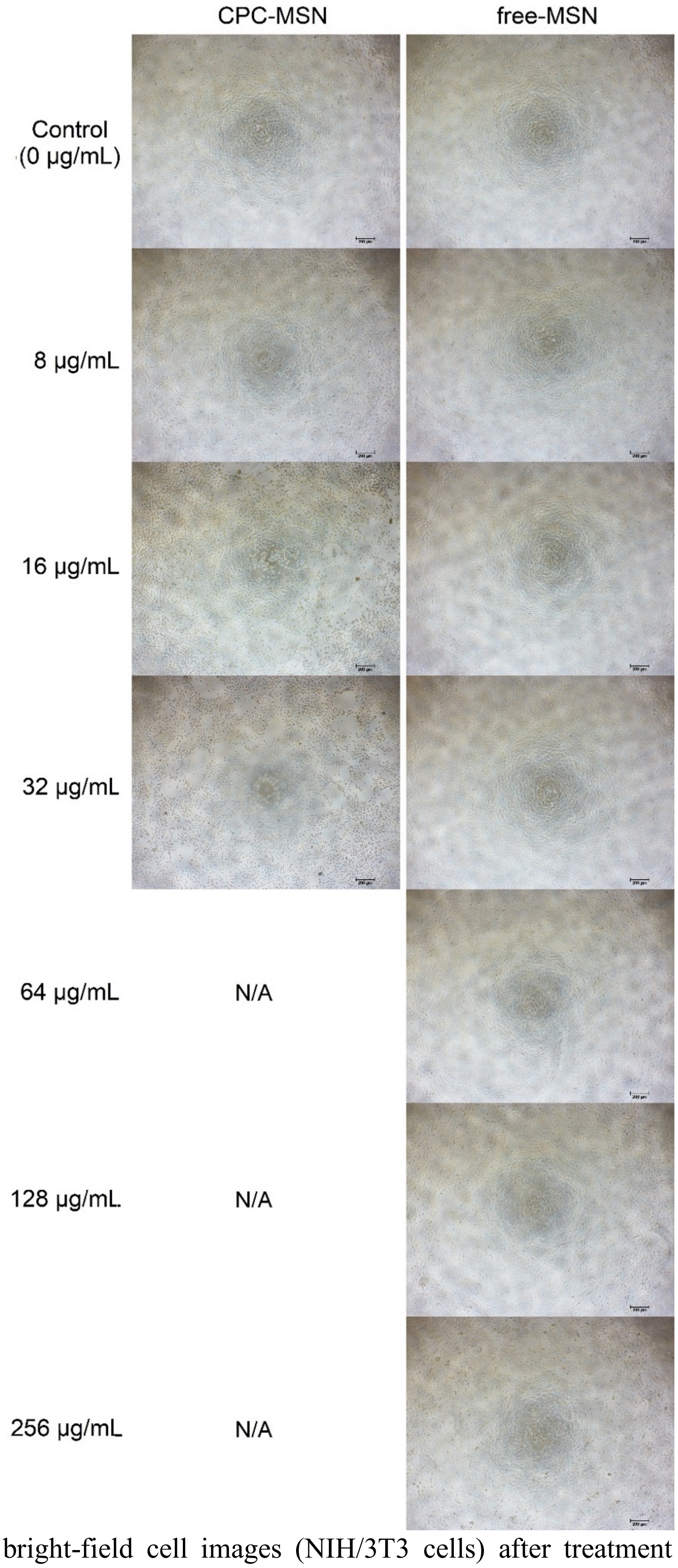

Figure S9. The bright-field cell images (NIH/3T3 cells) after treatment with **CPC-MSN** (loaded with CPC drug) 8–32 µg/mL and **free-MSN** (drug-free form) 8–256 µg/mL. Magnification ×4, scale bar = 200 µm.

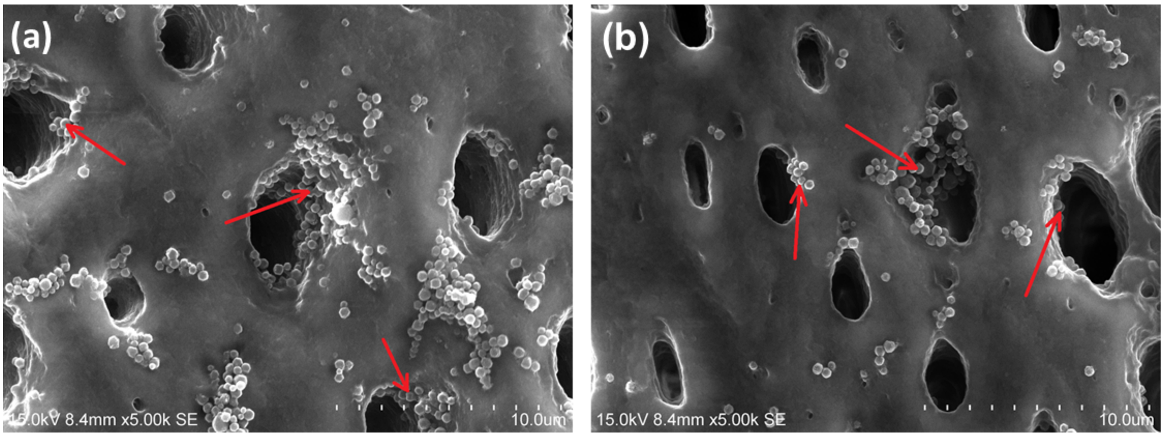

Figure S10. Additional scanning electron microscopic (SEM) images for dentinal tubule penetration by **CPC-MSN**. Arrows pointing to **CPC-MSN** inside dentinal tubules. Scale of (a) and (b) = 10 μm.

Table S1. NMR characterization of CPC drug used in this study:

^{1}H NMR	^{1}H NMR (400 MHz, $CDCl_3$, 298K) δ 9.47 (d, *J* = 5.6 Hz, 2H), 8.48 (t, *J* = 7.8 Hz, 1H), 8.12 (t, *J* = 7.1 Hz, 2H), 5.00 (t, *J* = 7.4 Hz, 2H), 2.05 – 1.96 (m, 2H), 1.38 – 1.15 (m, 26H), 0.86 (t, *J* = 6.9 Hz, 3H).
^{13}C NMR	^{13}C NMR (101 MHz, $CDCl_3$, 298K) δ 145.27, 145.16, 128.56, 62.08, 32.03, 31.94, 29.70, 29.57, 29.40, 29.15, 26.15, 22.71, 14.15.

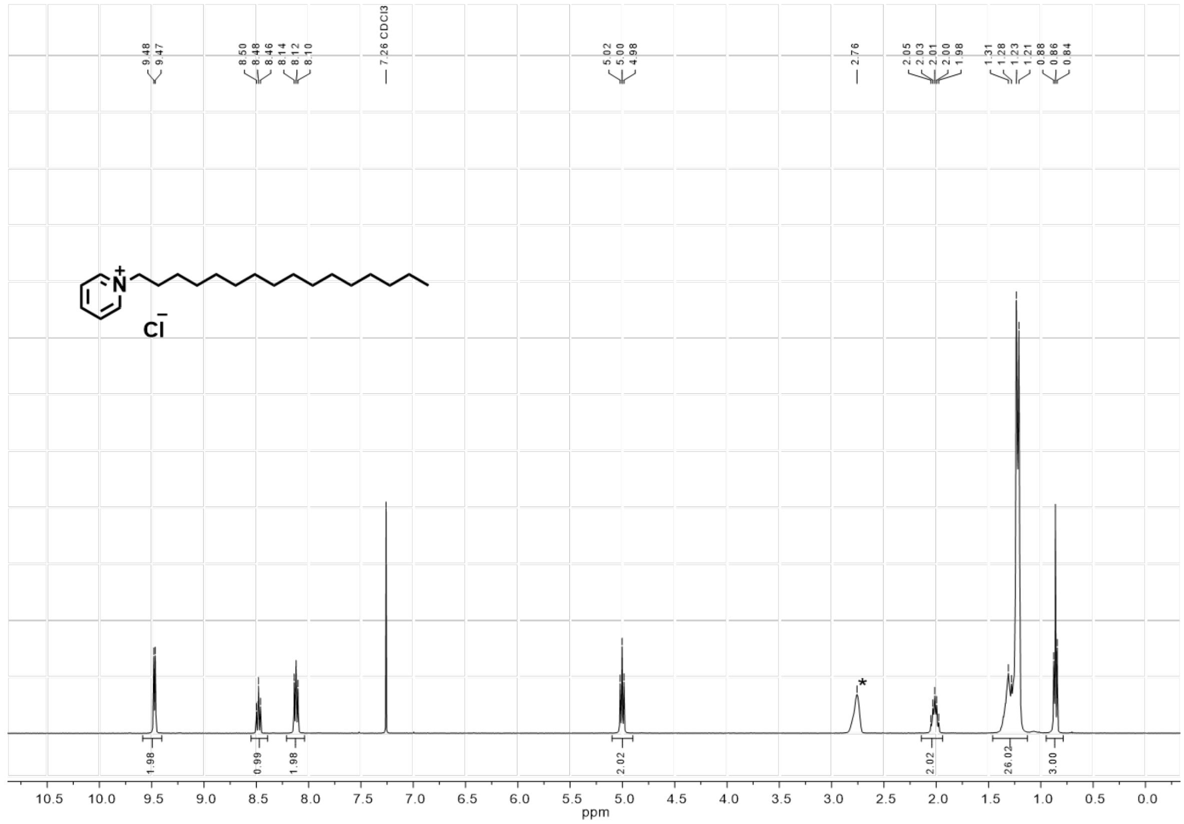

Figure S11. ^{1}H NMR spectrum (400 MHz, $CDCl_3$, 298 K) of pristine CPC used.

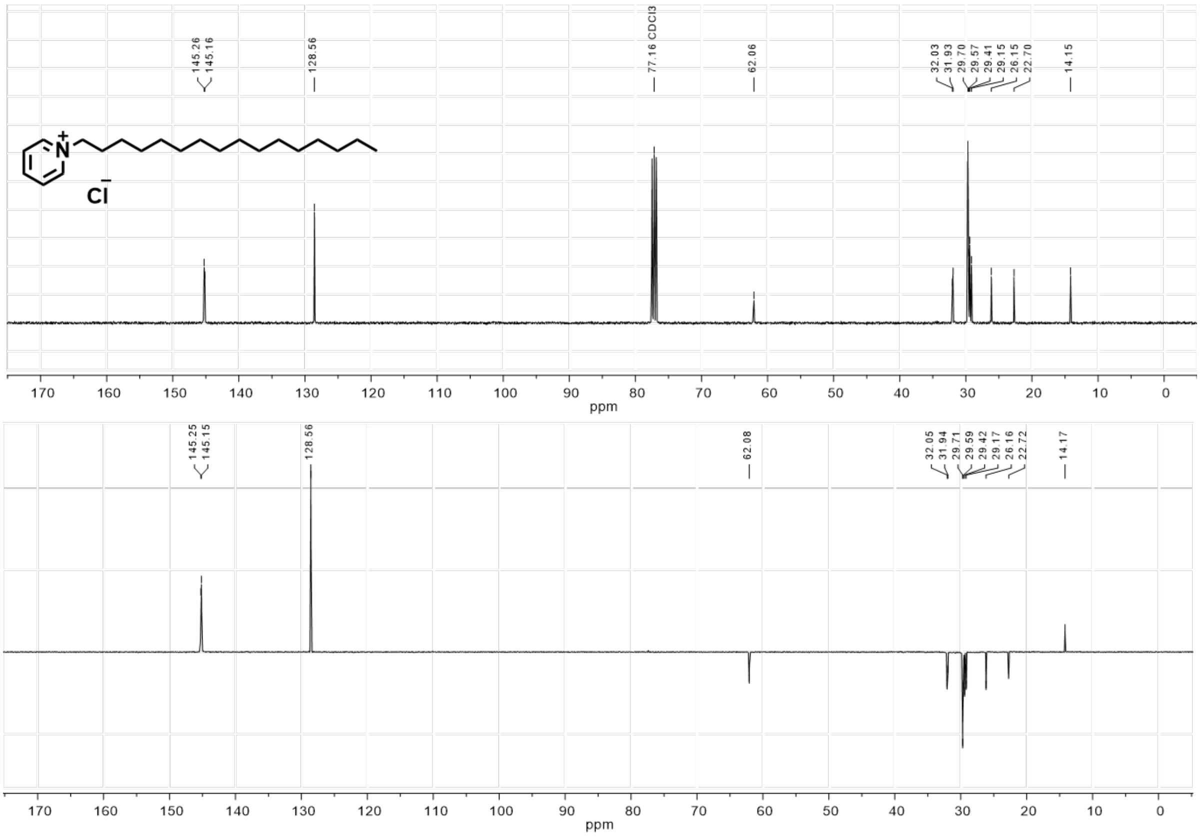

Figure S12. $^{13}C\{^{1}H\}$ NMR spectrum (101 MHz, $CDCl_3$, 298 K) of pristine CPC used.
